# *Trypanosoma cruzi* Phosphomannomutase and Guanosine Diphosphate-Mannose Pyrophosphorylase Ligandability Assessment

**DOI:** 10.1128/AAC.01082-19

**Published:** 2019-09-23

**Authors:** Filip Zmuda, Sharon M. Shepherd, Michael A. J. Ferguson, David W. Gray, Leah S. Torrie, Manu De Rycker

**Affiliations:** a Drug Discovery Unit, Wellcome Centre for Anti-Infectives Research, School of Life Sciences, University of Dundee, Dundee, United Kingdom; b Protein Production Team, Wellcome Centre for Anti-Infectives Research, School of Life Sciences, University of Dundee, Dundee, United Kingdom

**Keywords:** Chagas’ disease, drug discovery, ligandability, mannose, screening, target based

## Abstract

Chagas’ disease, which is caused by the Trypanosoma cruzi parasite, has become a global health problem that is currently treated with poorly tolerated drugs that require prolonged dosing. Therefore, there is a clinical need for new therapeutic agents that can mitigate these issues. The phosphomannomutase (PMM) and GDP-mannose pyrophosphorylase (GDP-MP) enzymes form part of the *de novo* biosynthetic pathway to the nucleotide sugar GDP-mannose. This nucleotide sugar is used either directly, or indirectly via the formation of dolichol-phosphomannose, for the assembly of all mannose-containing glycoconjugates. In T. cruzi, mannose-containing glycoconjugates include the cell-surface glycoinositol-phospholipids and the glycosylphosphatidylinositol-anchored mucin-like glycoproteins that dominate the cell surface architectures of all life cycle stages. This makes PMM and GDP-MP potentially attractive targets for a drug discovery program against Chagas’ disease. To assess the ligandability of these enzymes in T. cruzi, we have screened 18,117 structurally diverse compounds exploring drug-like chemical space and 16,845 small polar fragment compounds using an assay interrogating the activities of both PMM and GDP-MP enzymes simultaneously. This resulted in 48 small fragment hits, and on retesting 20 were found to be active against the enzymes. Deconvolution revealed that these were all inhibitors of T. cruzi GDP-MP, with compounds 2 and 3 acting as uncompetitive and competitive inhibitors, respectively. Based on these findings, the T. cruzi PMM and GDP-MP enzymes were deemed not ligandable and poorly ligandable, respectively, using small molecules from conventional drug discovery chemical space. This presents a significant hurdle to exploiting these enzymes as therapeutic targets for Chagas’ disease.

## Abstract (precis)

Chagas’ disease, which is caused by the Trypanosoma cruzi parasite, has become a global health problem that is currently treated with poorly tolerated drugs that require prolonged dosing. Therefore, there is a clinical need for new therapeutic agents that can mitigate these issues. The phosphomannomutase (PMM) and GDP-mannose pyrophosphorylase (GDP-MP) enzymes form part of the *de novo* biosynthetic pathway to the nucleotide sugar GDP-mannose.

## INTRODUCTION

Chagas’ disease is a vector borne disease, caused by the parasite Trypanosoma cruzi, with an increasing global health burden due to migration between Latin America, where it is endemic, and countries where it is not endemic. This is signified by the estimated 7 to 8 million people that are infected with T. cruzi worldwide, of which 300,000 are estimated to live in the United States and 59,000 to 108,000 are estimated to live in Europe (1). Infection with T. cruzi is first associated with an acute phase that tends to last up to approximately 8 weeks and, in most cases, patients present with nonspecific symptoms such inflammation at the site of parasite entry and fever. The acute phase usually resolves spontaneously, and most patients will enter an indeterminate chronic phase, where infection with T. cruzi persists in the absence of clinical symptoms and with good prognosis. However, approximately 30 to 40% of chronically infected patients will develop cardiac or gastrointestinal organ involvement over time, which can be fatal (2). For the last 4 decades, treatment of Chagas’ disease has been limited to benznidazole and nifurtimox, both of which are nitroheterocyclic drugs (3). Unfortunately, therapeutic failures are commonplace due to the long duration of treatments required with these drugs and their broad side effect profiles (2, 3), although the recent BENDITA clinical trial offers promise of a more effective and shorter-term treatment regimen (4). Despite this, there is a clear clinical need for new treatments against Chagas’ disease that are better tolerated and require shorter dosage regimens. One way to achieve this is through a focused drug discovery program targeting a validated biochemical pathway that is essential for parasite viability and/or virulence.

T. cruzi is known to produce an array of mannose-containing glycoconjugates, such as glycoinositol phospholipids (GIPLs) and glycosylphosphatidylinositol (GPI)-anchored mucin-like glycoproteins, trans-sialidase enzymes, and other N-glycosylated glycoproteins, which coat the outer surface of the parasite (5–8). Among other things, these glycoconjugates play an important role in parasite virulence and their ability to enter host cells (5–8). Importantly, genetic disruption of the biosynthetic pathway responsible for the production of similar glycoconjugate structures in related trypanosomatid parasites has been shown to be lethal to T. brucei (9–12) and to render Leishmania mexicana (13–16) and L. major (17) either less virulent or completely avirulent. A key intermediate in the production of these glycoconjugate structures is GDP-mannose (GDP-Man), which acts as a donor of activated mannose for glycosylation reactions. GDP-Man is known to be present at low basal levels in T. cruzi, T. brucei, and L. major, and this has been linked to the high flux of this substrate in trypanosomatids (6). This, coupled with the above-mentioned genetic findings, highlights the biological importance of GDP-Man in these parasites.

**FIG 1:**
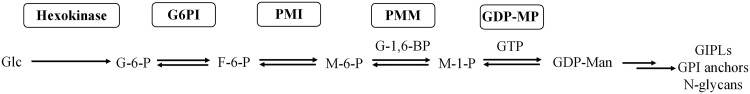
The GDP-Man biosynthetic pathway in T. cruzi. Glc, glucose; G-6-*P*, glucose-6-phosphate; G6PI, glucose-6-phosphate isomerase; F-6-*P*, fructose-6-phosphate; PMI, phosphomannose isomerase; M-6-*P*, mannose-6-phosphate; PMM, phosphomannomutase; G-1,6-BP, glucose-1,6-bisphosphate; M-1-*P*, mannose-1-phosphate; GDP-MP, GDP-mannose pyrophosphorylase; GDP-Man, GDP-mannose; GIPLs, glycosylinositol phospholipids; GPI, glycosylphosphatidylinositol.

In eukaryotes, GDP-Man is generated through a series of successive enzymatic reactions, where the penultimate step involves the conversion of mannose-6-phosphate (M-6-P) to mannose-1-phosphate (M-1-P) by the phosphomannomutase (PMM) enzyme through a transfer of a phosphate functionality, made available by the glucose-1,6-bisphosphate (G-1,6-BP) cofactor, from the C-6 position to the C-1 position of the mannose sugar (6, 17). It is important to note that this transformation is not associated with a mass change and cannot be measured directly using standard biochemical approaches in a high-throughput manner. The next step in this pathway involves the addition of M-1-P into the guanosine-monophosphate moiety of GTP by the GDP-mannose pyrophosphorylase (GDP-MP) enzyme to produce GDP-Man (6, 18) (Fig. 1). Drug discovery efforts utilizing high-throughput screening (19) and target-based design (20) approaches have been able to identify a small number of inhibitors of the L. mexicana and L. donovani GDP-MP enzymes that were capable of killing the parasites *in vitro*.

**FIG 2:**
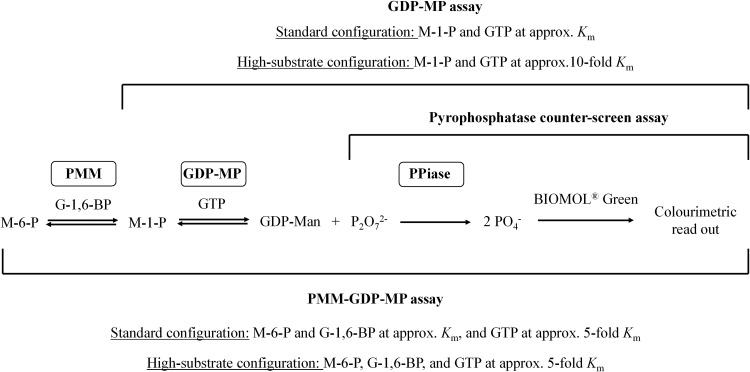
The biochemical assay platforms. M-6-*P*, mannose-6-phosphate; PMM, phosphomannomutase; G-1,6-BP, glucose-1,6-bisphosphate; M-1-*P*, mannose-1-phosphate; GDP-MP, GDP-mannose pyrophosphorylase; GDP-Man, GDP-mannose; PPiase, pyrophosphatase.

Collectively, these findings provide genetic and pharmacological evidence for the GDP-Man biosynthetic pathway as a target for new drugs against *Leishmania* and T. brucei parasites. However, the ability of T. cruzi PMM and GDP-MP to bind small molecules other than substrates *in vitro*, defined as “ligandability” (21), remains an open question. In order to address this knowledge gap, we have developed a high-throughput, colorimetric PMM and GDP-MP enzyme assay system to measure the collective output of both enzymes and a GDP-MP assay that measures the output of the single enzyme as a hit deconvolution strategy. Moreover, both assay platforms were further expanded to allow for interrogation of enzyme activities at different substrate concentrations (i.e., standard and high-substrate configurations) (Fig. 2). These tools were used to screen a subset of our diverse compound libraries and a focused small polar fragment library in an effort to identify chemical matter capable of inhibiting T. cruzi PMM and/or GDP-MP and subsequently further profile the pharmacology of hits of interest. Ultimately, the findings from these studies were used to assess the ligandability of the target T. cruzi PMM and GDP-MP enzymes.

## RESULTS

### Biochemical assay development.

**FIG 3:**
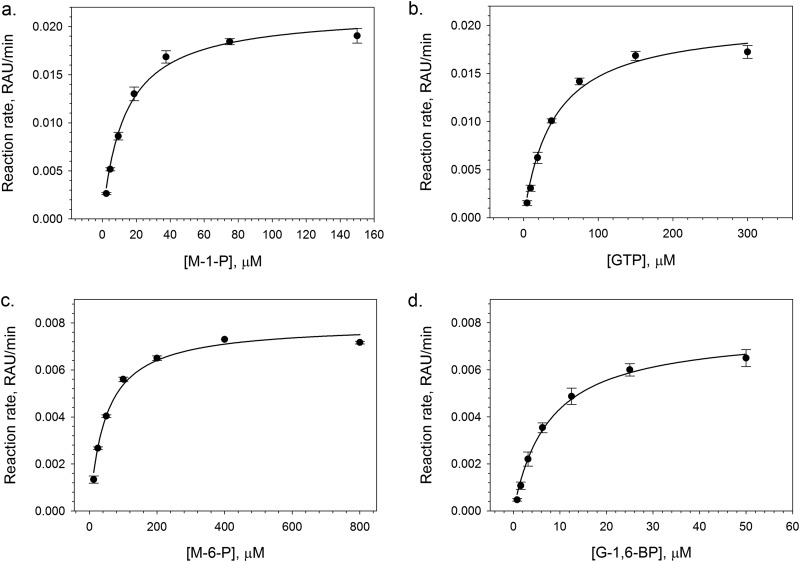
Michaelis-Menten plots for M-1-P (a) and GTP (b) substrates of the T. cruzi GDP-MP enzyme obtained using the GDP-MP biochemical assay, and the M-6-P substrate (c) and G-1,6-BP cofactor (d) of the T. cruzi PMM enzyme obtained using the PMM-GDP-MP biochemical assay. Data represents mean of three independent replicates (*n* = 3). Error bars represent ± the SD.

To identify suitable screening substrate concentrations, Michaelis-Menten constants (*K_m_*) were acquired for M-1-P and GTP substrates using the GDP-MP assay, followed by M-6-P and G-1,6-BP using the PMM-GDP-MP assay (Fig. 3 and Table 1). Multiple configurations of the assays were set up for screening and/or further pharmacological assessment using final substrate concentrations either approximately equivalent to *K_m_* values (“standard configurations”) or 5- to 10-fold *K_m_* values (“high-substrate configurations”). The linearity of both the GDP-MP and PMM-GDP-MP assays was investigated using the standard and high-substrate configurations. In the case of the GDP-MP assay, a linear response for up to 50 min (*R*^2^ = 0.98 and 0.99, respectively) was observed using both standard and high-substrate configurations (see Fig. S1a in the supplemental material). The PMM-GDP-MP assay biochemical response was associated with an initial lag phase of 30 min for both assay configurations, followed by a subsequent linear response for the remaining 60 min of the standard configuration time course reaction (*R*^2^ = 0.99). The lag phase was likely due to the need to build up M-1-P substrate by the PMM enzyme for the successive GDP-MP biochemical reaction. Time-course assay linearity was slightly shorter for the high-substrate assay configuration (i.e., 30 to 80 min; *R*^2^ = 0.99). Finally, as our screening compound libraries are formulated in dimethyl sulfoxide (DMSO), the tolerance of the biochemical assays to this solvent were investigated. Both the GDP-MP and the PMM-GDP-MP biochemical assays were shown to be tolerant to DMSO up to 2% (vol/vol) (Fig. S2).

**TABLE 1:** *K_m_* parameters for the T. cruzi GDP-MP substrates M-1-P and GTP, the T. cruzi PMM substrate M-6-P and the cofactor G-1,6-BP, and the substrate and cofactor concentrations that were chosen for screening using the standard and high-substrate assay configurations^*a*^

*K_m_* determination			Screening conditions, concn (μM)			
			Standard configuration		High-substrate configuration	
Substrate	*K_m_* (μM)^*a*^	95% CI (μM)	GDP-MP	PMM-GDP-MP	GDP-MP	PMM-GDP-MP
M-1-P	13.3	12.8–13.8	15		150	
GTP	40.7	31.9–51.6	30	150	300	150
M-6-P	48.2	44.3–52.4		45		225
G-1,6-BP	7.8	6.4–9.5		6		30

a The data represent the mean of three independent replicates (*n* = 3).

The biochemical assays rely on the detection of pyrophosphate using a colorimetric reporter system comprising of a pyrophosphatase enzyme that generates free inorganic phosphate, which in turn reacts with the BIOMOL Green reagent to generate a quantitative colorimetric response. To identify compounds capable of interfering with the pyrophosphatase and BIOMOL Green reporter, a counterscreen assay was established looking at this system in isolation. A titration of sodium pyrophosphate in the presence of a fixed concentration of pyrophosphatase reporter enzyme, which matched that used for the GDP-MP and PMM-GDP-MP biochemical assays, revealed complete turnover of pyrophosphate in less than 1 min (Fig. S3a). Importantly, the measured response signal was linear (*R*^2^ = 0.99) relative to pyrophosphate concentration (Fig. S3b).

### Diversity screening and hit confirmation.

**FIG 4:**
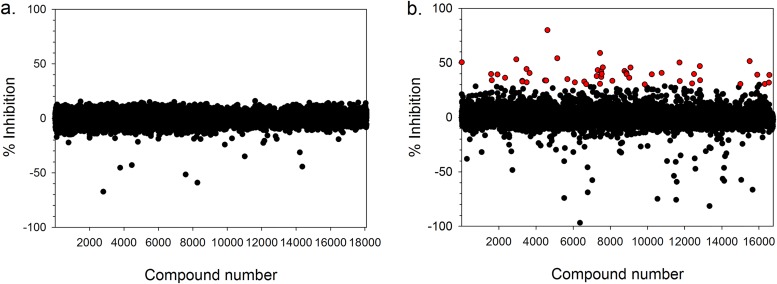
(a) Single point screen of 18,117 compounds at a concentration of 10 μM from two diversity compound sets and (b) single point screen of 16,845 compounds at a concentration of 300 μM from a small polar fragment set using the standard configuration T. cruzi PMM-GDP-MP biochemical assay. Red circles represent compounds exhibiting ≥30% inhibition. Interfering compounds exhibiting less than −100% inhibition were removed.

Single point screening of a diverse set of 18,117 compounds using the standard configuration PMM-GDP-MP assay platform failed to identify any chemical matter capable of inhibiting the biochemical activity of these enzymes by 30% or more at a compound concentration of 10 μM (Fig. 4a). However, screening a compound library comprising 16,845 small polar fragments at a higher compound concentration of 300 μM identified 48 hits at ≥30% inhibition (0.29% hit rate) (Fig. 4b). Mean robust Z′ values ± the standard deviations (SD) of 0.87 ± 0.04 (*n* = 60) and 0.90 ± 0.03 (*n* = 52) were obtained for the former and latter screens, where a Z′ of >0.5 signifies a highly robust biochemical assay (22).

**FIG 5:**
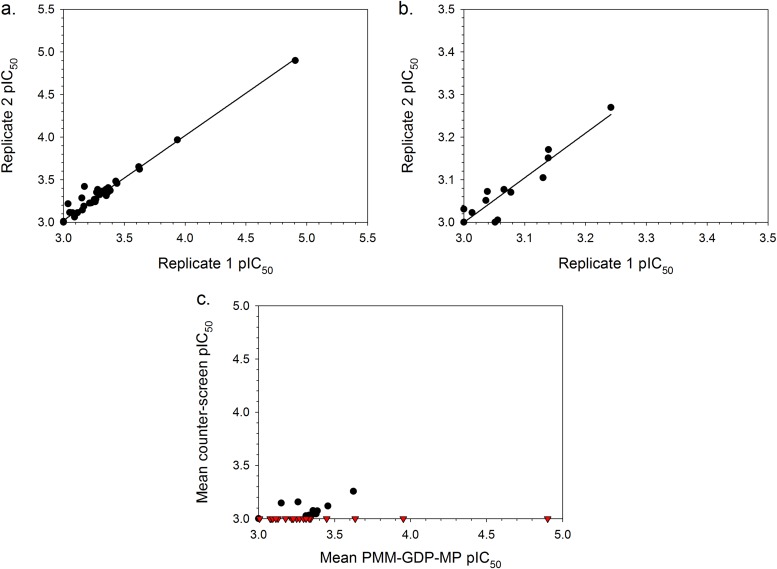
(a and b) Plots showing the linear correlations between pIC_50_ values of 46 hit compounds obtained for two independent replicate data sets acquired using the standard configuration T. cruzi PMM-GDP-MP biochemical assay (*R*^2^ = 0.98) (a) and the reporter technology interference counterscreen assay (*R*^2^ = 0.93) (b). (c) Plot of the mean pIC_50_ values (*n* = 2) for the hit compounds obtained using the standard configuration T. cruzi PMM-GDP-MP biochemical assay versus the reporter technology interference counterscreen assay. Red triangles represent 20 compounds that were active against the former assay (pIC_50_ ≥3) and inactive against the counterscreen assay (pIC_50_ <3).

**FIG 6:**
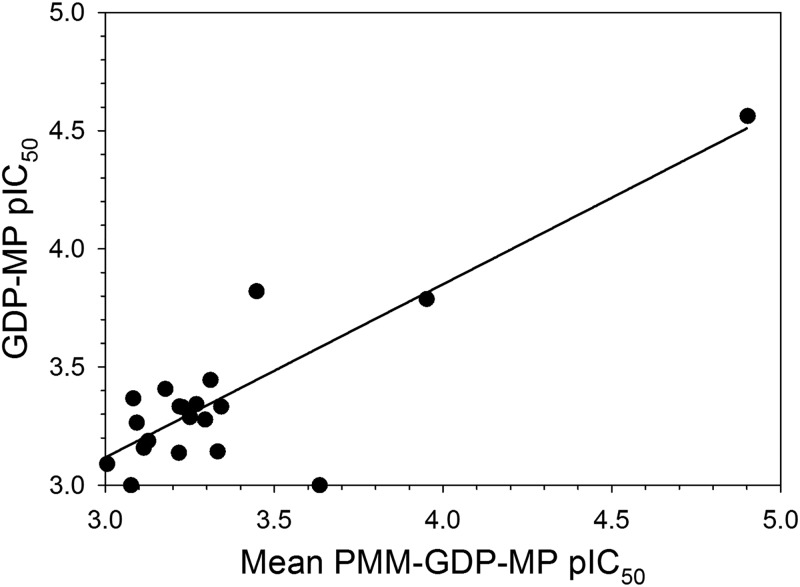
Plot showing the linear correlation (*R*^2^ = 0.81) between the mean pIC_50_ values (*n* = 2) of the 20 hit compounds obtained using the standard configuration T. cruzi PMM-GDP-MP assay versus the pIC_50_ values (*n* = 1) obtained using the standard configuration T. cruzi GDP-MP assay.

In order to establish the potency and confirm the presence or absence of technology interference, hit compounds that were available (46 of the 48) underwent concentration-response assessment using the standard configuration PMM-GDP-MP and reporter counterscreen biochemical assays. Excellent linear correlations were established between the calculated pIC_50_ parameters, defined as –log[IC_50_(M)], for independent assay replicates (Fig. 5a and b). Of the tested initial hit compounds, inhibitory activity (i.e., mean pIC_50_ of ≥3 [*n* = 2]) against the GDP-MP and/or PMM enzymes was established for 20 compounds, while the remaining compounds were either inactive (i.e., 13 compounds with mean pIC_50_ of <3 [*n* = 2]) or were deemed to be technology interferers (i.e., 13 compounds with mean pIC_50_ of ≥3 [*n* = 2] in the counterscreen) (Fig. 5c). To deconvolute the enzymatic target of the 20 hits, a concentration-response assessment was also performed using the standard configuration GDP-MP enzymatic assay (Fig. 6). Of the 20 hits, compound 1 exhibited much greater potency in both the PMM-GDP-MP and GDP-MP assays compared to the remaining compounds (Fig. 6 and Table 2). However, this compound has been identified as a hit against a number of other enzymes in our previous screening efforts and was deemed to be a promiscuous inhibitor. Compound 2 appeared to be active in the standard configuration PMM-GDP-MP assay but inactive in the standard configuration GDP-MP assay (Fig. 6 and Table 2), suggesting inhibitory activity against the PMM enzyme. To confirm the activity of compounds 2 and 3, fresh solid material was obtained, and both demonstrated activity in the standard and high-substrate configurations of the PMM-GDP-MP assay (Table 2; Fig. S4a and c in the supplemental material). In line with the poor level of enzyme inhibition, compounds 1 to 4 were inactive in our intracellular T. cruzi phenotypic assay (23) at 100 μM. Interestingly, compound 2 was shown to be inactive against the GDP-MP enzyme when tested using the standard configuration assay but capable of inhibition when substrate concentrations were increased by 10-fold (i.e., using the high-substrate configuration assay) (Table 2 and Fig. S4b), indicative of uncompetitive inhibition. The repurchased stock of compound 3 was also confirmed to be active in the standard configuration GDP-MP assay (Table 2 and Fig. S4d), with an observable shift in the concentration response curve and a subsequent drop in the pIC_50_ parameter when the high-substrate configuration of the assay was used, indicative of competitive inhibition.

**TABLE 2:**
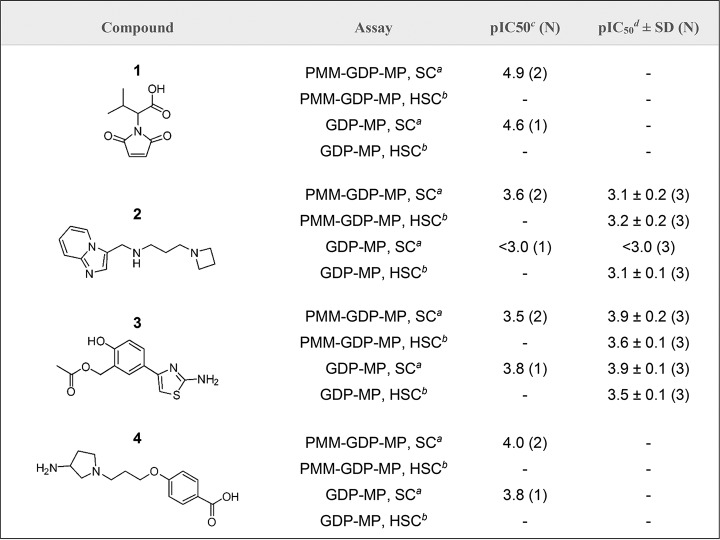
Structures and pIC_50_ parameters of the four most potent compounds (i.e., compounds 1 to 4) acquired using the standard configuration and high-substrate configuration T. cruzi PMM-GDP-MP and GDP-MP assays^*a*^

a Superscript notes: *a*, SC = standard configuration; *b*, HSC = high-substrate configuration; *c*, acquired using library stock material; *d*, acquired using repurchased material. The mean pIC_50_ parameter values are reported where *n* ≥ 2. Compounds with a pIC_50_ of <3.0 were deemed inactive in the corresponding biochemical assay.

## DISCUSSION

With the appropriate biochemical tools in place, we sought to address the knowledge gap of T. cruzi PMM and GDP-MP enzyme ligandability. Our initial efforts to identify inhibitors of either the T. cruzi PMM or the GDP-MP enzymes by performing a screen of 18,117 compounds exploring a diverse range of chemical space using the standard configuration PMM-GDP-MP assay platform were not successful (Fig. 4a). On the whole, the active sites of the PMM and GDP-MP enzymes exhibit a high level of homology between species (17, 20, 24) and *Leishmania* GDP-MP enzymes have been found to be ligandable by drug-like compounds. Lackovic et al. reported an initial hit rate of approximately 0.6% following a screen of approximately 80,000 compounds against L. major GDP-MP. After confirmation and technology interference counterscreening and hit expansion, the authors identified 21 inhibitors with cell-free pIC_50_ values spanning a range of 5.0 to 6.3. The majority of these compounds were classified into three distinct chemotypes, namely, pyrazolin-3,5-diones, 4-pyrizinylquinolines, and thiadiazole-like compounds (19). In addition to this, Mao et al. detailed 4 further inhibitors with p*K_i_* values ranging from 4.6 to 5.2 against L. mexicana and L. donovani GDP-MP enzymes (20). None of the previously published compounds were part of the screening sets used here. The screens did include a number of compounds with 4-pyrizinylquinoline and thiadiazole-like cores, which were all inactive in the standard configuration PMM-GDP-MP assay. Since no *Leishmania* enzyme data are available for these specific compounds, this information does not provide insight into the differences in ligandability between the *Leishmania* and T. cruzi enzymes.

It is possible that the apparent lower level of ligandability of T. cruzi PMM and/or GDP-MP compared to the *Leishmania* enzymes is due to subtle structural differences between the parasite species, which may drive the observed marked differences in the kinetic properties of the different GDP-MP enzymes. Mao et al. observed that the catalytic efficiency of the L. mexicana GDP-MP enzyme for M-1-P and GTP substrates was 10- and 20-fold higher, respectively, than the equivalent L. donovani enzyme (20). Moreover, our GTP *K_m_* value for T. cruzi GDP-MP was 2.4-, 5.3-, and 27-fold higher than reported for L. donovani, L. mexicana, and L. major, respectively, while the T. cruzi M-1-P *K_m_* parameter value was in line with the reported *Leishmania K_m_* values (19, 20).

By taking into account the polar nature of the substrates and/or cofactors for the PMM and GDP-MP enzymes, we attempted to bias our hunt for inhibitors of these T. cruzi enzymes by performing a screen using a focused compound library comprising of 16,845 small polar molecules. The use of lower molecular weight compounds for screening campaigns is usually associated with the identification of hits exhibiting weak inhibitory properties, which is an inherent consequence of the reduced number of potential interactions that can take place between a small fragment and the target enzyme (25). With this in mind, compounds from the small polar library were tested at a higher concentration of 300 μM compared to the previously screened compound sets. This endeavor was more successful as a small number of hits capable of inhibiting the biochemical response of the T. cruzi PMM-GDP-MP assay were identified. An initial deconvolution screen using the standard configuration GDP-MP assay showed that the majority of the compounds were also active in this assay, indicating that these were inhibitors of GDP-MP. The exception to this was compound 2 that showed marked activity against the standard configuration PMM-GDP-MP assay but appeared inactive in the standard configuration GDP-MP deconvolution assay, suggesting that compound 2 was an inhibitor of the T. cruzi PMM enzyme. However, on further concentration-response evaluation, compound 2 was found to display behavior that is consistent with uncompetitive GDP-MP enzyme inhibition, where the GTP substrate molecule must first be bound to the enzyme to allow binding of the inhibitory compound (26). This is supported by the similar pIC_50_ values obtained for compound 2 using the standard configuration PMM-GDP-MP and high-substrate configuration GDP-MP assays that both utilized the GTP substrate at saturating concentrations, and the lack of activity in the standard configuration GDP-MP assay where the GTP substrate was used at a concentration approximating its *K_m_* value. In contrast to this, compound 3 exhibited behavior that was suggestive of competitive inhibition of the T. cruzi GDP-MP enzyme, as exemplified by the observable shift in the dose-response curve and the corresponding decrease in the pIC_50_ value following a 10-fold increase in biochemical assay substrate concentrations.

In summary, our inability to identify and confirm a single inhibitor of the T. cruzi PMM enzyme from a total of 34,962 compounds exploring both drug-like and small polar compounds suggests that, in this particular chemical space, T. cruzi PMM is not ligandable. Moreover, based on criteria reported by Edfeldt et al. (21), the T. cruzi GDP-MP was deemed to be poorly ligandable as only a small number of polar fragment-like hits were identified, which exhibited weak inhibitory properties in our cell free biochemical assays. Therefore, despite genetic and pharmacological evidence for PMM and GDP-MP enzymes as potential therapeutic targets for leishmaniasis, the apparent poor ligandability of the T. cruzi variants presents an important hurdle for exploiting these targets for Chagas’ disease drug discovery.

## MATERIALS AND METHODS

### General.

All enzyme and substrate solution dilutions were prepared in assay buffer comprising 25 mM Tris-HCl (pH 7.5), 150 mM NaCl, 4 mM MgCl_2_, 0.01% (vol/vol) Tween 20, and 1 mM dithiothreitol in ultrapure water. Biochemical assays were performed in a 50-μl final assay volume using clear 384-well assay microplates (Greiner, catalog no. 781101) at room temperature. Unless stated otherwise, all reagent concentrations are written as final biochemical assay concentrations, and DMSO or compounds were added to assay plates using Echo acoustic dispensers (Labcyte). Reagent addition for high-throughput screening assays was performed using an Xrd-384 liquid dispenser (FluidX, UK) and a BioFill Solo/Xrd-384 16 channel resin nozzle (2.00 to 200 μl) tubing cartridge (FluidX, catalog no. 34-1005-S). Biochemical reactions were terminated by addition of 50 μl of BIOMOL Green (Enzo Life Sciences, catalog no. BML-AK111-1000) reagent, after which absorbance was measured following a 30-min incubation at room temperature. Absorbance measurements were performed at 650 nm using a PHERAStar microplate reader (BMG Labtech, Germany), and the data are represented as relative absorbance units (RAU). Linear and nonlinear regression was performed using the SigmaPlot 12.5 software, unless stated otherwise.

### *T. cruzi* PMM and GDP-MP production and purification.

The genes coding for T. cruzi GDP-MP and PMM (UniProtKB Q4CMK4 and Q4E4A3, respectively) were synthesized, followed by optimization of the codons for Escherichia coli by GenScript and cloning into modified pET15b with an N-terminal histidine and maltose-binding protein tag and tobacco etch virus (TEV) cleavage site for GDP-MP and an N-terminal histidine tag and TEV cleavage site for PMM. The respective plasmids were transformed into BL21(DE3) E. coli cells, and autoinduction media (1 liter for GDP-MP and 2 liters for PMM) supplemented with AMP was inoculated and grown for 4 h at 37°C, followed by a further 17 h at 20°C. Cell pellets were generated by centrifugation at 3,500 × *g* for 20 min, and in each case the supernatant was discarded. The pellets were resuspended in 20 ml of lysis buffer (i.e., 25 mM Tris [pH 8.5]; 500 mM NaCl; 20 mM imidazole; 1 cOmplete protease inhibitor Cocktail tablet [Roche, Germany]), and the resulting suspension was then passed through a continuous flow cell disruptor (Constant Systems, Ltd., UK) at 30,000 lb/in^2^. The T. cruzi GDP-MP sample was then centrifuged at 37,500 × *g* for 30 min and filtered (0.45-μm pore size), and the PMM sample was centrifuged at 40,000 × *g* for 30 min and filtered (0.22-μm pore size).

To purify the proteins, a 5-ml HisTrap Ni HP column (GE Healthcare) was first equilibrated with buffer A (i.e., 25 mM Tris [pH 8.5], 500 mM NaCl, 20 mM imidazole) using an ÄKTA pure chromatography system (GE Healthcare), followed by loading of protein sample at 5 ml/min. Next, the column was washed with 10 column volumes of buffer A, followed by 10 column volumes of 5% (vol/vol) buffer B (i.e., 25 mM Tris [pH 8.5], 500 mM NaCl, 0.5 mM TCEP, 500 mM imidazole) to remove histidine-rich contaminating proteins. A gradient of 5% (vol/vol) to 50% (vol/vol) buffer B was then used to elute the proteins. TEV protease was added to the protein samples (7 mg for GDP-MP and 2 mg for PMM), which were then dialyzed against buffer C (i.e., 25 mM Tris [pH 7.5] and 250 mM NaCl). The proteins were passed through the 5-ml HisTrap Ni HP column (GE Healthcare) to remove the TEV protease and any uncleaved proteins, where cleaved proteins were retained on the column. A gradient of 0% (vol/vol) to 50% (vol/vol) of buffer B over 10 column volumes was applied to remove the cleaved protein from the histidine and/or maltose binding protein, and TEV. Protein samples were concentrated to 11 ml using 30-kDa cutoff Vivaspin protein concentrators (Sartorius, Germany), filtered (0.22-μm pore size), and loaded onto XK26/60 Superdex 200 and 75 columns (GE Healthcare) for GDP-MP and PMM, respectively, which were previously equilibrated with buffer C. Protein loading was performed using a 10-ml loop at 1 ml/min. The GDP-MP and PMM proteins were eluted using buffer C as hexamers and dimers, respectively (columns were calibrated with Bio-Rad [USA] standards). T. cruzi GDP-MP was concentrated to 2.49 mg/ml, and a total yield of 12 mg was achieved. T. cruzi PMM was concentrated to 5.77 mg/ml, and a total yield of 3.24 mg was achieved. Mass spectrometry was used to confirm the identity of the proteins, and densitometry (measured using a Bio-Rad imager) was used to confirm that both protein samples exhibited 100% purity.

### Primary assay development.

To calculate the Michaelis constant (*K_m_*) for the M-1-P substrate, 20-min time course reactions using a fixed concentration of GDP-MP enzyme (3.13 nM), a saturating concentration of GTP substrate (600 μM) mixed with inorganic pyrophosphatase (1 U/ml; Merck, catalogue no. I5907-1MG), and various concentrations of M-1-P substrate (150, 75, 37.5, 18.8, 9.38, 4.69, and 2.34 μM) were performed. The *K_m_* for the GTP substrate was acquired in a similar manner using a saturating concentration of M-1-P (150 μM) and various concentrations of GTP substrate (300, 150, 75, 37.5, 18.8, 9.38, and 4.69 μM). In both cases, background control samples were also included that lacked the presence of GDP-MP enzyme. At the end of the time courses, the biochemical reactions were terminated, and absorbance measurements were taken. Background signal was subtracted and linear regression was performed on the corresponding time versus activity plots in order to acquire relative reaction rates, which were in turn used to generate classical Michaelis-Menten plots. Data were acquired from three independent replicates (*n* = 3) and are represented in the figures as mean values ± the SD. The *K_m_* was obtained by fitting individual replicate data to the Michaelis-Menten equation shown below, where v is the reaction velocity, [S] is the substrate concentration, and *V*_max_ is the maximum reaction velocity:

$$v=\frac{V_{\text{max}}\left[\text{S}\right]}{K_{m}+\left[\text{S}\right]}$$

To measure the activity of the T. cruzi PMM enzyme, a PMM-GDP-MP biochemical assay system was used. *K_m_* determinations for the M-6-P substrate and G-1,6-BP cofactor were acquired as described above by performing 60-min time course reactions. Fixed concentrations of PMM and GDP-MP enzyme (6.25 and 3.13 nM, respectively), a saturating concentration of GTP (300 μM) mixed with inorganic pyrophosphatase (1 U/ml), and a saturating concentration of either M-6-P (300 μM) or G-1,6-BP (60 μM) and various concentrations of either G-1,6-BP (i.e., 50, 25, 12.5, 6.25, 3.13, 1.57, and 0.78 μM) or M-6-P (i.e., 800, 400, 200, 100, 50, 25, and 12.5 μM) were used. Data were acquired from three independent replicates (*n* = 3) and are represented in the figures as mean values ± the SD.

To confirm the linearity of the GDP-MP assay in the standard and high-substrate configurations, a 50-min time course reaction was performed using either 3.13 or 0.78 nM GDP-MP enzyme, respectively, in the presence of M-1-P (15 or 150 μM), GTP (30 or 300 μM), and inorganic pyrophosphatase (1 U/ml). In a manner similar to that described above, the standard and high-substrate configurations of the PMM-GDP-MP assay were tested for linearity by performing 90-min time course reactions using 6.25 nM PMM and 3.13 nM GDP-MP enzymes in the presence of M-6-P (45 or 225 μM), G-1,6-BP (6 or 30 μM), GTP (150 μM), and inorganic pyrophosphatase (1 U/ml). In all cases, background control samples were also included that lacked the presence of enzyme. At the end of the time courses, the biochemical reactions were terminated, and absorbance measurements were taken. Data were acquired from four technical replicates (*n* = 4) for each enzyme and assay configuration and are represented as a mean RAU values ± the SD after subtraction of the background signal. Linear regression models were fitted to the complete data sets from the standard and high-substrate configurations of the GDP-MP assay, and for the 30- to 90-min and 30- to 80-min time frames for standard and high-substrate configurations of the PMM-GDP-MP assays, respectively.

### DMSO tolerance.

DMSO tolerance of the GDP-MP enzyme was investigated by incubating the enzyme (3.13 nM) in the presence of GTP (30 μM), M-1-P (15 μM), inorganic pyrophosphatase (1 U/ml), and various volumes of DMSO (i.e., 1,000, 500, 250, 125, 60, and 30 nl corresponding to 2, 1, 0.5, 0.25, 0.12, and 0.06% [vol/vol] final assay concentrations, respectively) at room temperature for 60 min. DMSO was added to the assay microplate using a Preddator liquid dispenser (Redd&Whyte, UK). Background control samples were also included that lacked the presence of enzyme. At the end of the incubation period, the biochemical reactions were terminated and absorbance measurements were taken. In a similar manner, DMSO tolerance of the PMM enzyme was investigated by incubating PMM and GDP-MP enzymes (6.25 and 3.13 nM, respectively) in the presence of GTP (150 μM), M-6-P (45 μM), G-1,6-BP (6 μM), and inorganic pyrophosphatase (1 U/ml). Data were acquired from four technical replicates (*n* = 4) for each enzyme and are represented in the figures as mean RAU values + the SD after subtraction of background signal.

### Pyrophosphatase counterscreen assay development.

In order to evaluate the activity of the inorganic pyrophosphatase reporter enzyme, a 10-minute time course reaction using a fixed concentration of the enzyme (1 U/ml) and various concentrations of sodium pyrophosphate substrate (20, 10, 5, 2.5, 1.25, and 0 μM) was performed. Background control samples were also included that lacked the presence of pyrophosphatase enzyme. At the end of the time course, the biochemical reactions were terminated and absorbance measurements were taken. Data were acquired from two technical replicates (*n* = 2) and are represented as background subtracted RAU. To confirm the linearity of the assay, the inorganic pyrophosphatase reporter enzyme (1 U/ml) was incubated at room temperature for 5 min in the presence of various concentrations of sodium pyrophosphate substrate (20, 15, 10, 7.5, 5, 3.75, 2.50, 1.88, 1.25, and 0 μM). The colorimetric reaction was developed, and the data were acquired as described above. Data were obtained from five technical replicates (*n* = 5) and are represented as mean RAU values ± the SD after the subtraction of background signal. A linear regression model was fitted to the resulting plot.

### Single-point diversity screening.

Single-point diversity screening was performed using the standard configuration PMM-GDP-MP biochemical assay and microplates containing compounds in columns 1 to 22 from two diverse small-molecule compound sets covering traditional small-molecule chemical space (DDD, 9,257 compounds; molecular weights between 185.2 and 492.2) and more three-dimensional chemical space (GHCDL, 8,860 compounds; molecular weights between 190.24 and 449.3) at a final concentration of 10 μM (final DMSO concentration = 0.1% [vol/vol]) or a small polar set (16,845 primarily fragment-sized compounds with increased polarity; molecular weights between 88.1 and 476.0) at a final concentration of 300 μM (final DMSO concentration = 0.3% [vol/vol]). Columns 23 and 24 both contained DMSO (either 0.1 or 0.3% [vol/vol] at final assay concentration) and were utilized as “maximum effect” and “minimum effect” control populations, respectively. For screening purposes, 25 μl of a solution containing PMM and GDP-MP enzymes (6.25 and 3.13 nM, respectively) was added to columns 1 to 23, and 25 μl of buffer only was added to column 24. This was followed by the addition of 25 μl of a mixture containing inorganic pyrophosphatase (1 U/ml) and substrates/cofactors (150 μM GTP, 45 μM M-6-P, and 6 μM G-1,6,-BP). The assay plates were incubated at room temperature for 90 min. At the end of the incubation period, the biochemical reactions were terminated, and absorbance measurements were taken. Robust Z′ values were calculated for each assay microplate using the following equation:

$$\begin{array}{l} \text{Robust Z}’=\\ 1–\left(\begin{array}{l} \left(3\times \left(1.483\times \left(\text{RAU MAD Max}\right)\right)\right)+\\ \left(3\times \left(1.483\times \left(\text{RAU MAD Min}\right)\right)\right) \end{array}\right)/\left(\text{median RAU Max}-\text{ median RAU Min}\right) \end{array}$$

where MAD is the median absolute deviation, Max are the maximum effect control samples, and Min are the minimum effect control samples.

RAU values were normalized to percent inhibition values relative to the maximum and minimum effect control populations, and compounds displaying percent inhibition values of ≥30% were selected as hits. Data were processed using IDBS ActivityBase 8.1.2.12 and Dotmatics Limited Vortex v2017.08.69598-59-s software.

### Concentration-response assays.

Concentration-response assessment of hits using the PMM-GDP-MP assays was performed by adding 25 μl of a solution of PMM and GDP-MP enzymes (6.25 and 3.13 nM, respectively) to columns 1 to 11 and 13 to 23 of microplates containing various concentrations of compounds (10 final assay concentrations ranging from 990 to 1.92 μM at 1 in 3 dilution increments in the presence of 1% [vol/vol] DMSO). Buffer only (25 μl) was added to columns 12 and 24. Columns 11 and 23 were utilized as “maximum effect” control populations (1% [vol/vol] DMSO with enzyme), and columns 12 and 24 were “minimum effect” control populations (1% [vol/vol] DMSO in the absence of enzyme). This was followed by the addition of 25 μl of a mixture containing inorganic pyrophosphatase (1 U/ml) and substrates/cofactors (150 μM GTP, 45 μM M-6-P, and 6 μM G-1,6-BP for the standard configuration assay and 150 μM GTP, 225 μM M-6-P, and 30 μM G-1,6-BP for the high-substrate configuration assay) to the entire assay plate. The standard configuration assay plates were incubated at room temperature for 90 min, and the high-substrate configuration assay plates were incubated for 80 min. Standard and high-substrate configuration GDP-MP assay compound concentration-response assessments were performed as described above by incubating GDP-MP enzyme (3.13 or 0.78 nM) in the presence of GTP (30 or 300 μM), M-1-P (15 or 150 μM), and inorganic pyrophosphatase (1 U/ml) for 50 min at room temperature. Pyrophosphatase counterscreen concentration-response assessments were performed by preincubating compounds with inorganic pyrophosphatase (1 U/ml) for 90 min at room temperature, followed by the addition of inorganic pyrophosphate (10 μM) and a secondary 5-min incubation at room temperature. At the end of the incubation periods, the biochemical reactions were terminated and absorbance measurements were taken. RAU values were normalized to % inhibition values relative to the maximum and minimum effect control populations. pIC_50_ values, defined as –log[IC_50_(M)], were calculated by fitting concentration-response data for each independent replicate separately to a four-parameter logistic model, as shown below, using either the IDBS ActivityBase 8.1.2.12 or SigmaPlot 12.5 software. Where appropriate, the top curve plateau parameter was constrained to a value of 100. Compounds requiring a concentration greater than 990 μM to inhibit biochemical assay activity by at least 50% were deemed to be inactive (i.e., pIC_50_ <3).

The four-parameter logistic model was as follows, where Min is the minimum asymptote and Max is the maximum asymptote:

$$y=\text{Min}+\frac{\text{Max – Min}}{1+{\left(\frac{10^{-{\text{logIC}}_{50}}}{x}\right)}^{\text{Hill slope}}}$$

## Supplementary Material

Supplemental file 1
