# Supplementary material for: Trypanosoma cruzi Phosphomannomutase and Guanosine Diphosphate-Mannose Pyrophosphorylase Ligandability Assessment
Source: Antimicrob Agents Chemother. 2019 Sep 23;63(10):e01082-19. doi: 10.1128/AAC.01082-19 (PMC6761512; doi:10.1128/AAC.01082-19)
Supplement: Supplemental file 1 [file AAC.01082-19-s0001.pdf]

***Trypanosoma cruzi* phosphomannomutase and guanosine diphosphate-mannose pyrophosphorylase ligandability assessment.**

SUPPORTING INFORMATION

Filip Zmuda[a], *Sharon M. Shepherd*[b], *Michael A. J. Ferguson*[a], *David. W. Gray*[a#], *Leah S. Torrie* [a], *Manu De Rycker*[a#]

[a]Drug Discovery Unit, Wellcome Centre for Anti-Infectives Research, School of Life Sciences, University of Dundee, Dow Street, Dundee DD1 5EH, United Kingdom.

[b]Protein Production Team, Wellcome Centre for Anti-Infectives Research, School of Life Sciences, University of Dundee, Dow Street, Dundee DD1 5EH, United Kingdom.

Table of Contents

Linearity of *T. cruzi* GDP-MP and PMM-GDP-MP standard and high-substrate configuration biochemical assays. S2

DMSO tolerance of T. cruzi GDP-MP and PMM-GDP-MP biochemical assays. S2

Reporter counter-screen assay substrate titration time-course and assay linearity. S3

Concentration response curves for re-purchased compounds **2** and **3**. S4

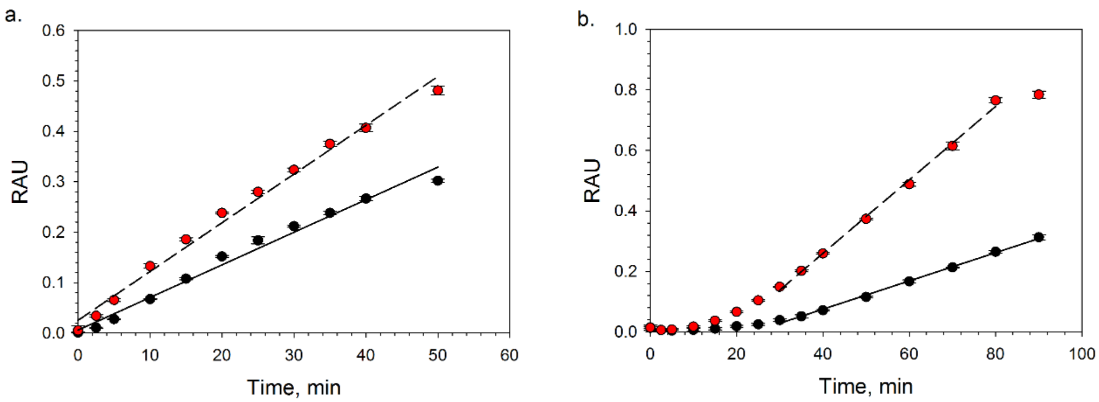

Figure S1. (a) *T. cruzi* GDP-MP biochemical assay time-course for the standard (black circles and solid line; R^2 = 0.98) and high-substrate configurations (empty circles and dashed line; R^2 = 0.98). (b) *T. cruzi* PMM-GDP-MP biochemical assay time-course for the standard (black circles and solid line; 30–90 minute R^2 = 0.99) and high-substrate configurations (red circles and dashed line; 30–80 minute R^2 = 0.99). Data represents mean of 4 technical replicates (N = 4). Error bars represent ± SD.

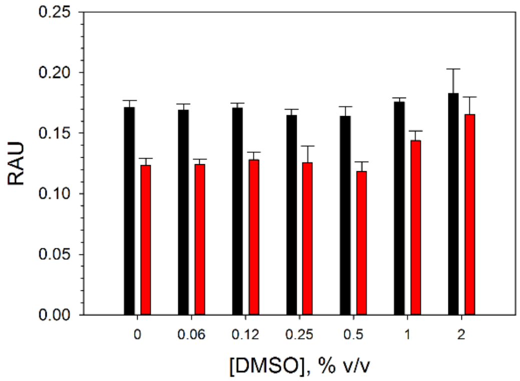

Figure S2. Tolerance of the standard configuration *T. cruzi* GDP-MP (black bars) and standard configuration PMM-GDP-MP (red bars) assays to varying concentrations of DMSO. Data represents mean of 4 technical replicates (N = 4). Error bars represent + SD.

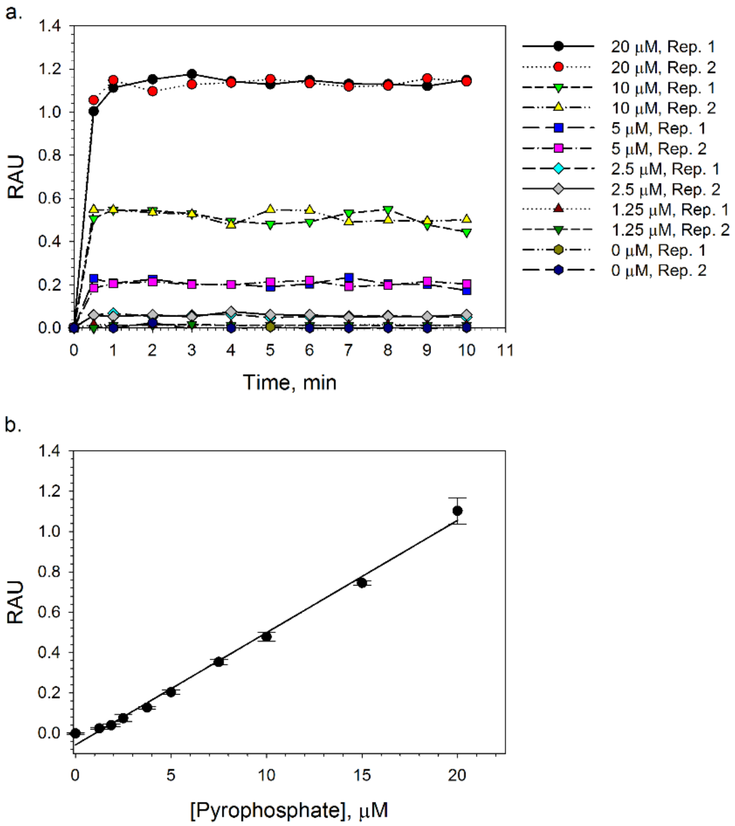

Figure S3. (a) Reporter counter-screen biochemical assay 10 minute time-course in the presence of varying concentrations of sodium pyrophosphate. Data shown for two technical replicates (i.e. Rep. 1 and Rep. 2). (b) Reporter counter-screen biochemical assay linearity at a fixed biochemical reaction time of 5 minutes. Data represents mean of 5 technical replicates (N = 5). Error bars represent ± SD; R^2 = 0.99.

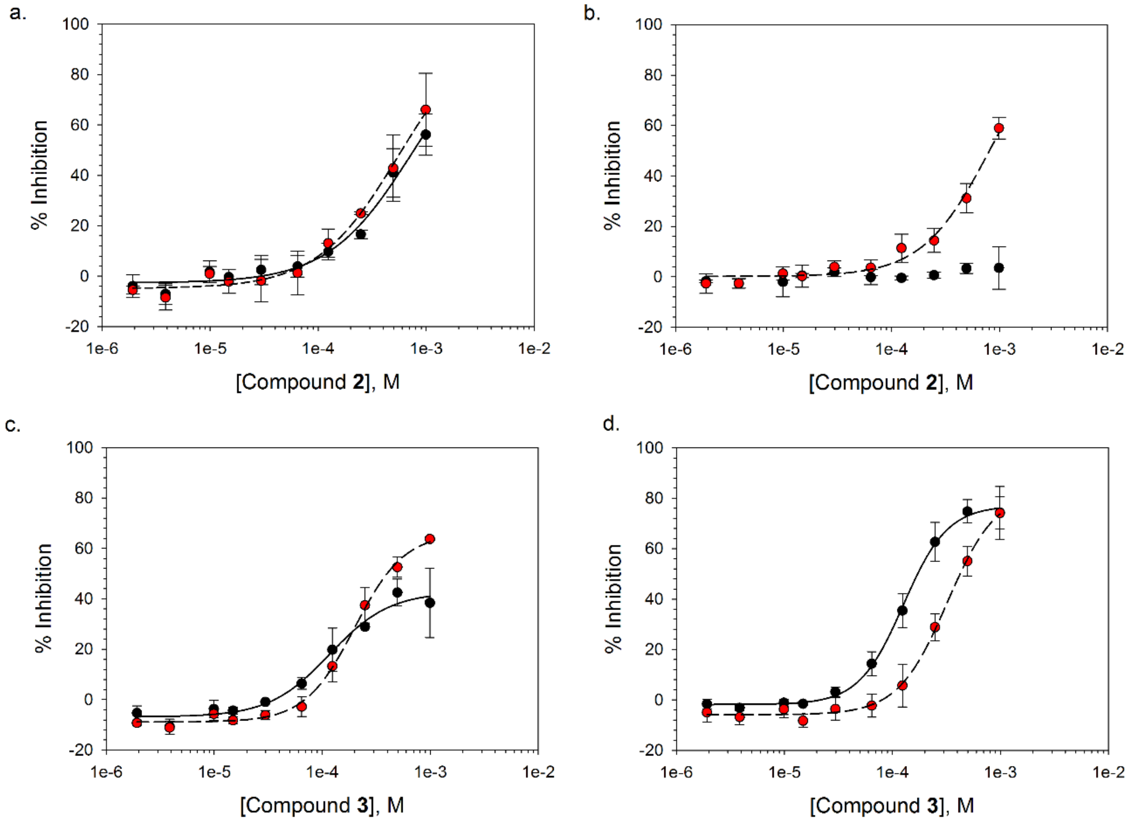

Figure S4. Concentration-response plots for compounds **2** and **3** acquired using the *T. cruzi* PMM-GDP-MP (a and c) or GDP-MP (b and d) assays. Black circles and solid lines represent the standard configuration assays, and red circles and dashed lines represent the high-substrate configuration assays. Data represents mean of three independent replicates (N = 3). Error bars represent ± SD.
